# Metronidazole Causes Skeletal Muscle Atrophy and Modulates Muscle Chronometabolism

**DOI:** 10.3390/ijms19082418

**Published:** 2018-08-16

**Authors:** Ravikumar Manickam, Hui Yun Penny Oh, Chek Kun Tan, Eeswari Paramalingam, Walter Wahli

**Affiliations:** 1 Lee Kong Chian School of Medicine, Nanyang Technological University, 11 Mandalay Road, Singapore 308232, Singapore; ravikumar_vet@hotmail.com (R.M.); HOH001@e.ntu.edu.sg (H.Y.P.O.); willcktan@hotmail.com (C.K.T.); eeswari28@hotmail.com (P.E.); 2 Interdisciplinary Graduate School, NTU Institute for Health Technologies, Nanyang Technological University, 50 Nanyang Avenue, Singapore 639798, Singapore; 3 Center for Integrative Genomics, University of Lausanne, Le Génopode, CH-1015 Lausanne, Switzerland

**Keywords:** metronidazole, gut dysbiosis, skeletal muscle atrophy, circadian rhythm

## Abstract

Antibiotics lead to increased susceptibility to colonization by pathogenic organisms, with different effects on the host-microbiota relationship. Here, we show that metronidazole treatment of specific pathogen-free (SPF) mice results in a significant increase of the bacterial phylum *Proteobacteria* in fecal pellets. Furthermore, metronidazole in SPF mice decreases hind limb muscle weight and results in smaller fibers in the tibialis anterior muscle. In the gastrocnemius muscle, metronidazole causes upregulation of *Hdac4*, *myogenin*, *MuRF1*, and *atrogin1*, which are implicated in skeletal muscle neurogenic atrophy. Metronidazole in SPF mice also upregulates skeletal muscle *FoxO3*, described as involved in apoptosis and muscle regeneration. Of note, alteration of the gut microbiota results in increased expression of the muscle core clock and effector genes *Cry2*, *Ror*-*β*, and *E4BP4*. *PPARγ* and one of its important target genes, *adiponectin*, are also upregulated by metronidazole. Metronidazole in germ-free (GF) mice increases the expression of other core clock genes, such as *Bmal1* and *Per2*, as well as the metabolic regulators *FoxO1* and *Pdk4*, suggesting a microbiota-independent pharmacologic effect. In conclusion, metronidazole in SPF mice results in skeletal muscle atrophy and changes the expression of genes involved in the muscle peripheral circadian rhythm machinery and metabolic regulation.

## 1. Introduction

The mammalian gut hosts the most diverse population of microorganisms, including bacteria, archaea, protozoa and fungi, and even viruses. The gut microbiota primarily provides protection against pathogenic microorganisms and inflammation and helps with digestion and energy metabolism by modulating various host signaling pathways [1]. The daily rhythmicity of the gut microbiota and its metabolites modulates the host circadian rhythm [2]. Furthermore, gut dysbiosis and circadian rhythm disruption are associated with metabolic syndrome and diseases such as obesity, type 2 diabetes, and inflammatory bowel disease [3].

The gut microbiota composition is modulated by diet and antibiotics [4,5]. Antibiotics alter microbiota composition and expression of microbial genes and derived metabolites such as bile acid products and short-chain fatty acids, methylamines, and indoles [4,6]. Alteration of gut microbiota composition may lead to deleterious effects on the host and colonization by antibiotic-resistant pathogens [7]. Most of these findings come primarily from studies in animals exposed to various antibiotics, including metronidazole (1-(2-hydroxyethyl)-2-methyl-5-nitroimidazle) for a period of 4 weeks [8,9]. 

Furthermore, comparative analyses of specific pathogen-free (SPF) and germ-free (GF) mice are often used to study the effects of microbiota on host metabolic and immune functions. Several studies have demonstrated that GF mice are protected from diet-induced obesity, low-grade inflammation, and glucose intolerance as compared to conventionally raised animals [10,11]. Furthermore, microbiota transfer via fecal transplantation evokes modifications in body weight and insulin sensitivity in both rodents and humans [4,10,12].

Metronidazole is in the nitroimidazole class of antimicrobial drugs, frequently used to treat anaerobic, protozoal, and *Helicobacter pylori* infections [13]. It causes transient loss of colonization resistance to pathogens [14]. When administered orally, metronidazole is absorbed almost completely and distributed widely within the body. It is extensively metabolized by the liver, most likely by the hepatic cytochrome P450 system, and its degradation products are excreted in urine and feces [13]. Metronidazole is believed to enter the cerebrospinal fluid and the central nervous system, which can cause neurotoxicity [13]. The effects in the central nervous system range from seizures to metronidazole-induced encephalopathy and cerebellar syndrome. Recently, antibiotic-associated encephalopathy was identified as a cause of delirium in patients receiving antibiotics including metronidazole; however, peripheral neuropathy is the most common adverse effect of metronidazole [15]. Of interest, many muscle functions, including energy metabolism, myokine secretion, tissue homeostasis, and regeneration, are influenced by the skeletal muscle peripheral clock and the clock-controlled genes [16,17,18], but the mechanism by which metronidazole causes muscle weakness is not known. Because the microbiota participates in the control of the liver peripheral clock [19], we hypothesized that, among other effects, metronidazole may also influence the skeletal muscle peripheral clock.

Here we demonstrate that metronidazole causes changes in the expression of genes implicated in the muscle peripheral clock machinery, metabolic regulators, and skeletal muscle neurogenic atrophy that might be associated with peripheral neuropathy, the most commonly observed adverse side effect. Metronidazole also causes protein degradation and thus acts as a double-edged sword in skeletal muscle atrophy.

## 2. Results

### 2.1. Metronidazole Enhances Susceptibility to Colonization by Proteobacteria and Enrichment of Erysipelotrichales

**Figure 1:**
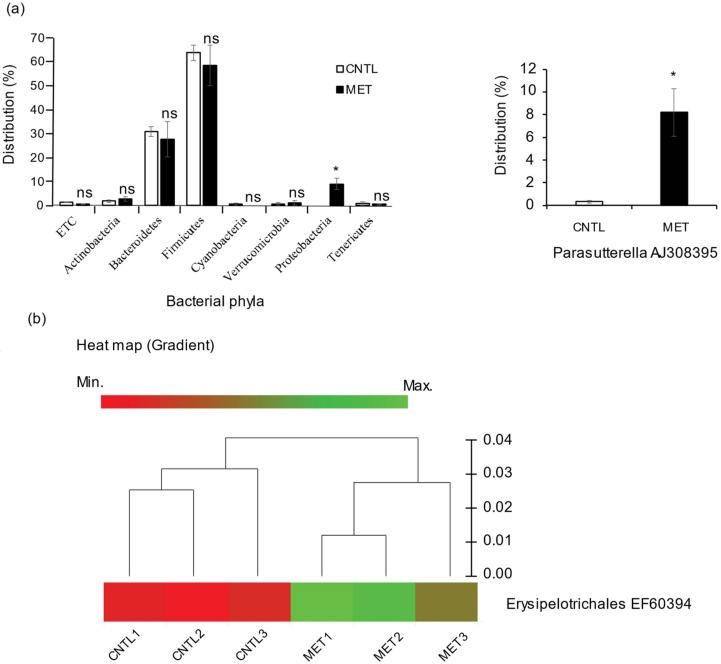
Metronidazole enhances susceptibility to colonization by *Proteobacteria* and enrichment of *Erysipelotrichales*. 16s rRNA genes were sequenced from DNA extracted from the fecal pellets of metronidazole (MET)-treated and nontreated control (CNTL) specific pathogen–free (SPF) mice. (**a**) Percentage representation of the bacterial phyla distribution in fecal pellets of metronidazole-treated and nontreated control SPF mice demonstrating increased susceptibility to colonization by *Proteobacteria*, especially *Parasutterella*. *N* = 3 mice per group. Data are presented as means ± standard error of the mean (SEM). Asterisks indicate statistically significant differences (*, *p* < 0.05; ns, nonsignificant with student’s *t* test). (**b**) Gradient heatmap showing enrichment of the bacterial species *Erysipelotrichales* in metronidazole-treated SPF mice as compared to nontreated controls. Relative abundance of the bacterial species is color coded from minimum (red) to maximum (green). *N* = 3 mice per nontreated control (CNTL1–3) and metronidazole-treated (MET1–3) groups.

Consistent with other publications [8,9], we found that metronidazole treatment had a marginal impact on the major bacterial phyla distribution in specific pathogen-free (SPF) mouse fecal pellets analyzed by 16S rRNA gene sequencing. However, we found a slight but significant increase in the susceptibility to colonization by *Proteobacteria*, especially *Parasutterella* AJ308395_s, compared to nontreated control mice (Figure 1a). Furthermore, there was substantial enrichment of the bacterial species *Erysipelotrichales* EF603943_s in the metronidazole-treated SPF mice when compared to the nontreated control mice (Figure 1b).

### 2.2. Metronidazole Causes Skeletal Muscle Atrophy

**Figure 2:**
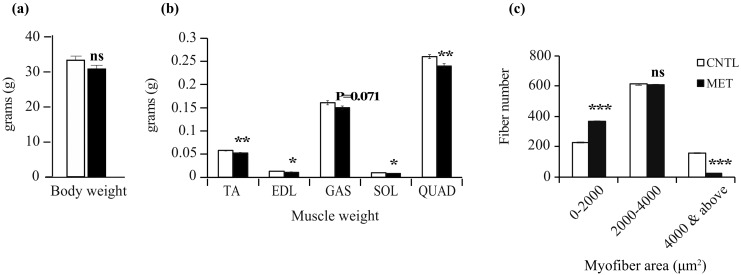
Metronidazole causes reduced hind limb muscle weight and myofiber surface area. (**a**) Metronidazole treatment of SPF mice resulted in a marginal, nonsignificant decrease in body weight compared to nontreated controls. *N* = 5 mice per group. (**b**) Absolute hind limb muscle weights of tibialis anterior (TA), extensor digitorum longus (EDL), gastrocnemius (GAS), soleus (SOL), and quadriceps (QUAD) muscles of metronidazole-treated SPF mice compared to nontreated controls. *N* = 5 mice per group. (**c**) Myofiber numbers according to myofiber surface area in the tibialis anterior in metronidazole-treated and nontreated SPF mice. *N* = 3 mice per group. A thousand random myofibers per mouse were counted across the mid-belly region of the tibialis anterior muscle cryosections. Data presented as means ± SEM of 3000 myofibers per group (CNTL and MET). Asterisks indicate statistically significant differences (*, *p* < 0.05; **, *p* < 0.01; ***, *p* < 0.001; ns, nonsignificant with student’s *t* test).

The body weight of metronidazole-treated SPF mice was marginally but not significantly reduced when compared to nontreated control mice (Figure 2a). However, the body weight of metronidazole-treated germ-free (GF) mice was significantly reduced when compared to nontreated control mice (Supplementary Figure S1a). The weight of the hind limb muscles of metronidazole-treated SPF and GF mice was significantly reduced compared to nontreated control mice, with the exception of the soleus in GF mice (Figure 2b and Supplementary Figure S1b). Noticeably, the relative weight of the hind limb muscles of both SPF and GF mice did not display any significant differences between metronidazole-treated and nontreated control mice (data not shown). However, histological examination of the tibialis anterior muscle at the mid-belly region of the metronidazole-treated SPF mice revealed a significant decrease in larger myofibers and a concomitant increase in smaller myofibers compared to nontreated control mice (Figure 2c). Taken together, these data suggest that metronidazole treatment might lead to skeletal muscle atrophy.

### 2.3. Metronidazole Causes Skeletal Muscle Neurogenic Atrophy

**Figure 3:**
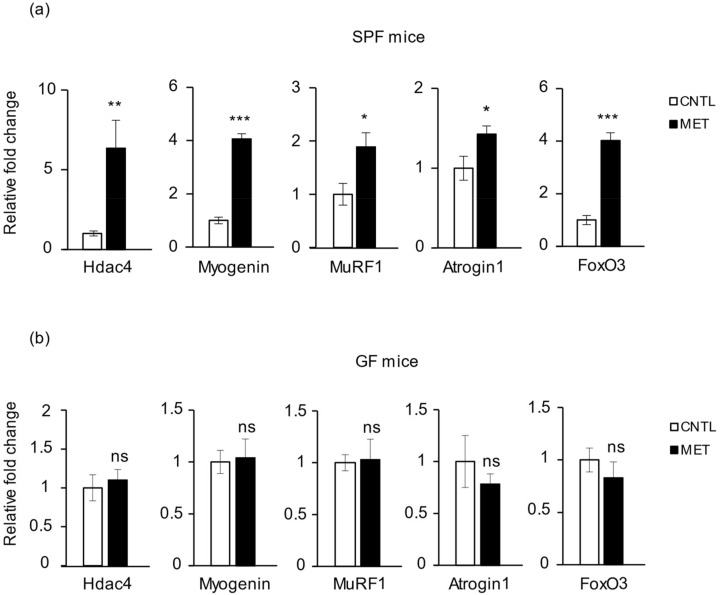
Metronidazole causes changes in the expression of skeletal muscle atrophy genes. Real-time quantitative PCR analysis of *Hdac4*, *myogenin*, *MuRF1*, *atrogin1*, and *FoxO3* expression in gastrocnemius of metronidazole-treated and nontreated (**a**) SPF and (**b**) GF mice. *N* = 5 mice per group. Data presented as means ± SEM. Asterisks indicate statistically significant differences (*, *p* < 0.05; **, *p* < 0.01; ***, *p* < 0.001; ns, nonsignificant with student’s *t* test).

Denervation of skeletal muscle upregulates the histone deacetylases 4 and 5 (Hdac4/5), which then repress dachshund family transcription factor 2 (Dach2), a negative regulator of *myogenin*. Increased myogenin in turn upregulates the ubiquitin E3 ligases MuRF1 and atrogin1, causing skeletal muscle atrophy [20]. Our analysis of gene expression in the mouse gastrocnemius revealed a significant upregulation of skeletal muscle neurogenic atrophy markers, such as *Hdac4*, *myogenin*, *MuRF1*, and *atrogin1* expression, in metronidazole-treated SPF mice as compared to nontreated control animals (Figure 3a). This effect was not observed in GF mice treated with metronidazole (Figure 3b). The expression level of *Hdac5* did not differ in SPF mice treated with and without metronidazole (Supplementary Figure S2a). *Dach2* expression level was reduced in metronidazole-treated SPF mice when compared to nontreated control mice (Supplementary Figure S2a), but this trend did not reach statistical significance (*p* = 0.063).

Forkhead box protein O3 (FoxO3) is a key transcription factor in protein breakdown, because it modulates the activity of several actors in the ubiquitin–proteasome and autophagy–lysosomal proteolytic pathways, including mitochondrial autophagy, also known as mitophagy [21,22]. Analysis of mouse gastrocnemius revealed a significant increase in *FoxO3* expression level in SPF mice treated with metronidazole when compared to nontreated control mice, and the effect was not observed in GF mice (Figure 3a,b). Furthermore, expression levels of the master regulator gene of myogenesis, *MyoD*, the muscle mass–determining dominant-negative gene *myostatin*, and the autophagy marker genes *Mul1* and *LC3-1* were unaffected by metronidazole treatment in SPF mice compared to nontreated control mice (Supplementary Figure S2b). Together, these data suggest that metronidazole causes skeletal muscle atrophy through upregulation of *myogenin* and *FoxO3*.

### 2.4. Metronidazole Is Involved in Skeletal Muscle Energy Metabolism

**Figure 4:**
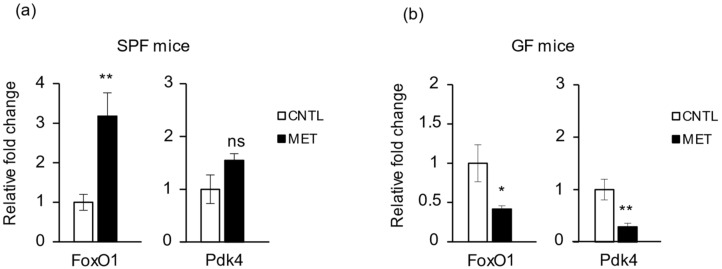
Metronidazole affects the expression of skeletal muscle metabolism genes. Real-time quantitative PCR analysis of *FoxO1* and *Pdk4* expression in gastrocnemius of metronidazole-treated and nontreated (**a**) SPF and (**b**) GF mice. *N* = 5 mice per group. Data presented as means ± SEM. Asterisks indicate statistically significant differences (*, *p* < 0.05; **, *p* < 0.01; ns, nonsignificant with student’s *t* test).

FoxO1 is a transcription factor that plays an important role in glucose metabolism by insulin signaling. In skeletal muscle, FoxO1 increases expression of the mitochondrial pyruvate dehydrogenase (acetyl-transferring) kinase isozyme 4 (Pdk4). This kinase phosphorylates subunits of pyruvate dehydrogenase, which inhibits the activity of the enzyme. Inhibition of pyruvate dehydrogenase decreases glucose use and increases fat metabolism, as during fasting [23,24]. We found that *FoxO1* expression in gastrocnemius was significantly upregulated in SPF mice treated with metronidazole compared to nontreated control mice (Figure 4a). Of note, metronidazole treatment of GF mice triggered an opposite effect, with a significant reduction in expression level of *FoxO1* compared to nontreated control GF mice (Figure 4b). As a FoxO1 target gene, *Pdk4* was upregulated in SPF mice treated with metronidazole (not significantly, however, likely because of individual variation) and significantly downregulated, together with *FoxO1*, in GF mice treated with metronidazole (Figure 4b). Furthermore, there was no change in the expression levels of metabolic regulators such as *Sirt1*, *Ampk*, *Pgc*-*1α*, *PPARα*, and *PPARβ/δ* in SPF mice treated with metronidazole compared to nontreated control animals (Supplementary Figure S2c).

Together, these data suggest that, through its impact on *FoxO1* and *Pdk4* expression in SPF mice, metronidazole could decrease glucose use in skeletal muscle through an effect on the microbiota, which may participate with other effects in the observed skeletal muscle atrophy. In contrast, in the skeletal muscle of GF mice, metronidazole had a microbiota-independent pharmacologic effect that would promote glucose use.

### 2.5. Metronidazole Modulates Expression of Muscle Core Clock and Clock-Controlled Genes

**Figure 5:**
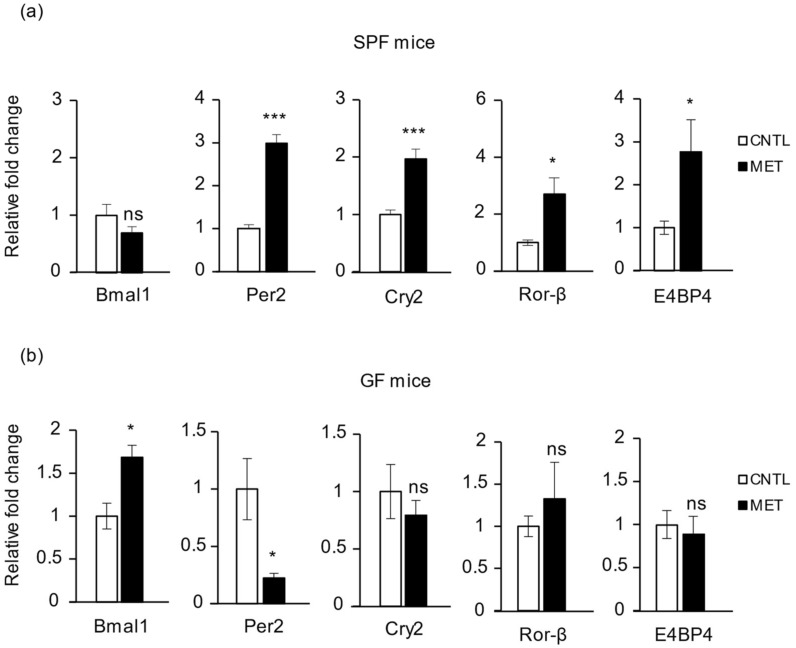
Metronidazole alters skeletal muscle core clock and clock effector gene expression. Real-time quantitative PCR analysis of *Bmal1*, *Per2*, *Cry2*, *Ror*-*β*, and *E4BP4* expression in gastrocnemius of metronidazole-treated and nontreated (**a**) SPF and (**b**) GF mice. *N* = 5 mice per group. Data presented as means ± SEM. Asterisks indicate statistically significant differences (*, *p* < 0.05; ***, *p* < 0.001; ns, nonsignificant with student’s *t* test).

Transcription of the clock gene *Cry1* follows a circadian pattern in skeletal muscle [25], and drugs such as prednisolone and ramipril can modulate skeletal muscle clock genes [26,27]. The gastrocnemius muscles of metronidazole-treated SPF mice displayed significant upregulation of the core clock genes *Per2* and *Cry2* and a downregulation trend for *Bmal1* when compared to nontreated control mice (Figure 5a). Furthermore, *Ror*-*β* and *E4BP4* were also significantly upregulated after metronidazole treatment (Figure 5a). In contrast, the metronidazole-treated GF mice displayed significant upregulation of *Bmal1* and downregulation of *Per2* as compared to the nontreated control GF mice, again indicating a microbiota-independent effect opposite to that of the microbiota-dependent effect in SPF mice (Figure 5b). Moreover, expression levels of *Clock*, *Per1*, *Cry1*, *Ror*-*α* and *Ror*-*γ*, and *Rev*-*erbα* and *Rev*-*erbβ* did not differ significantly, and neither did those of clock-controlled genes *Dbp* and *Dec2* in the SPF mice treated with metronidazole (Supplementary Figure S2d).

**Figure 6:**
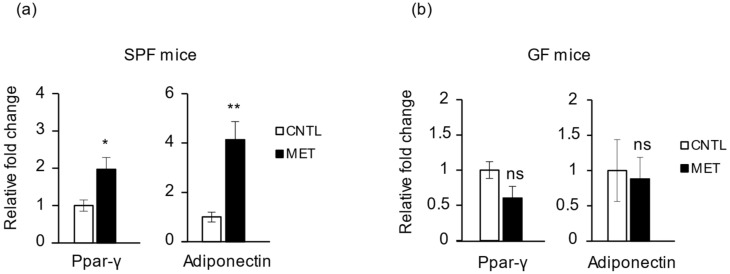
Metronidazole modulates skeletal muscle *adiponectin* and *PPARγ* expression. Real-time quantitative PCR analysis of *PPARγ* and *adiponectin* expression in gastrocnemius muscle of metronidazole-treated and nontreated (**a**) SPF (**a**) and (**b**) GF mice. *N* = 5 mice per group. Data presented as means ± SEM. Asterisks indicate statistically significant differences (*, *p* < 0.05; **, *p* < 0.01; ns, nonsignificant with student’s *t* test).

Of note, *PPARγ* and its target gene *adiponectin* [28,29] were significantly upregulated in the SPF mice treated with metronidazole (Figure 6a), an effect that was absent in metronidazole-treated GF mice (Figure 6b). The expression of other factors important in metabolic regulation, such as *Sirt1*, *Ampk*, *Pgc*-*1α*, *PPARα*, and *PPARβ/δ*, was not affected by metronidazole in SPF mice (Supplementary Figure S2c). Together, these data show that in SPF mice, metronidazole affects some, but not all, genes of the muscle clock and genes important in metabolism. In GF mice, metronidazole also affects clock genes, but in a way opposite to that observed in SPF mice.

### 2.6. Metronidazole Stimulates Expression of RNA Modification of N^6^-Methyladenosine Complex Genes

**Figure 7:**
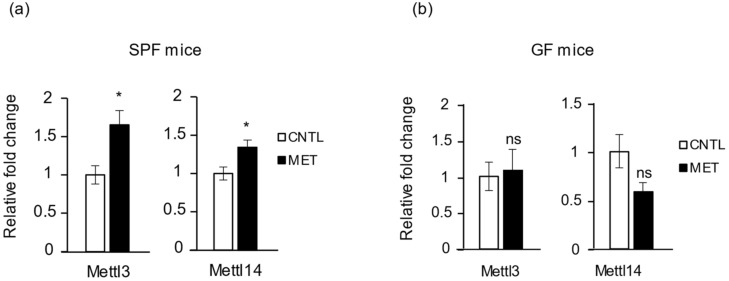
Metronidazole disrupts skeletal muscle RNA epigenetics. Real-time quantitative PCR analysis of RNA m^6^A methyltransferases *Mettl3* and *Mettl14* expression in gastrocnemius muscle of metronidazole-treated and nontreated (**a**) SPF and (**b**) GF mice. *N* = 5 mice per group. Data presented as means ± SEM. Asterisks indicate statistically significant differences (*, *p* < 0.05; ns, nonsignificant with student’s *t* test).

RNA modification is now recognized as playing a role in many important biological functions. N^6^-methyladenosine (m^6^A) is an abundant modification of mRNA found in most eukaryotic RNAs [30]. m^6^A can be incorporated by a methyltransferase complex, whose core is a methyltransferase such as 3 (METTL3)/METTL14 heterodimer, in which METTL3 provides the catalytic activity [31]. m^6^A is recognized by YTH domain–containing proteins, which instruct different complexes to regulate RNA signaling pathways [30]. It was recently found that m^6^A modification by METTL3 stabilizes MyoD mRNA levels, thereby promoting skeletal muscle differentiation [32]. We therefore analyzed whether metronidazole treatment changes the expression of genes for the methylation complex (*Mettl3*, *Mettl14*) and for a YTH domain–recognition protein (*Ythdf2*), as well as that of the *Fto* gene, whose mRNA is m^6^A methylated. We found that *Mettl3* and *Mettl14* showed increased expression in SPF mice treated with metronidazole compared to nontreated controls (Figure 7a). This effect did not occur in GF mice (Figure 7b). Also, we found no influence of metronidazole on *Fto* and *Ythdf2* expression in SPF mice (Supplementary Figure S2e). Collectively, these results demonstrate that metronidazole affects the expression of genes involved in the RNA epigenetic modification machinery in skeletal muscle.

## 3. Discussion

The goal of this study was to characterize the effects of metronidazole on gut dysbiosis and muscle health. We treated SPF and GF mice for 4 weeks with metronidazole in drinking water, similar to most published studies involving antibiotic-treated mice [8,9]. In this study, all mice had *ad libitum* access to food and water. We found that metronidazole caused gut dysbiosis with increased susceptibility to colonization by *Proteobacteria* and enrichment of *Erysipelotrichales* in the treated SPF mice. A recent report suggested that an increase in *Parasutterella* is associated with the development and progression of irritable bowel syndrome (IBS) and chronic inflammation of the intestine [33]. Transferring aged gut microbiota to young GF mice led to an increase in *Proteobacteria* involved in inflammatory aging and inflammation of the small intestine [34]. Similarly, an increase in *Erysipelotrichales* has been associated with inflammatory aging in older mice when compared to young mice [35]. Here we demonstrate that this dysbiosis correlates with skeletal muscle atrophy with upregulation of *Hdac4*, *myogenin*, and *FoxO1/O3*. It is possible that the increase in *myogenin* and *FoxO1/O3* stimulated expression of the E3 ubiquitin ligase genes *MuRF1* and *atrogin1* in the SPF mice treated with metronidazole. Therefore, metronidazole might act as a double-edged sword in skeletal muscle atrophy, causing skeletal muscle neurogenic atrophy through the upregulation of *Hdac4* and *myogenin* and *FoxO1/O3*-mediated protein degradation, resulting in the observed decrease in larger myofibers and consequent reduced hind limb muscle weight. The effect of metronidazole on skeletal muscle neurogenic atrophy genes in the SPF mice might explain the peripheral neuropathy observed in patients treated with metronidazole. We can speculate that the endotoxins produced by metronidazole-resistant proteobacteria might initiate a tumor necrosis factor α–mediated inflammatory response, inhibiting Akt signaling and upregulating the *FoxO* target genes *MuRF1*, *atrogin1*, and *Pdk4* [36]. Moreover, the upregulation of *Pdk4* in SPF mice treated with metronidazole may inactivate the pyruvate dehydrogenase complex, which is rate-limiting in muscle carbohydrate oxidation [37].

Furthermore, our results demonstrate a novel effect of metronidazole on genes involved in the control of muscle circadian rhythm, energy metabolism, and RNA epigenetic modifications. The gut microbiota and its daily rhythmicity of produced metabolites is reported to modulate host circadian chromatin dynamics and transcriptome in intestine and liver [2]. It was proposed that microbiota-generated signals are integrated by peripheral tissues to temporally organize genome-wide transcription [2]. The mammalian circadian clock is a transcriptional and translational negative feedback loop controlled by the core clock gene products Clock, Bmal1, Per1-3, and Cry1 and Cry2, eventually regulating many biological processes [38]. The peripheral clocks in different organs are coordinated by both the central master clock located in the suprachiasmatic nucleus of the hypothalamus and zeitgebers such as light/dark, feeding/fasting, and rest/activity cycles [39]. The skeletal muscle peripheral clock and the clock-controlled genes have been implicated in energy metabolism, myokine secretion, regeneration, and tissue homeostasis [16,17,18]. Our results show that alteration of the microbiota composition with metronidazole in mice affects the host’s circadian rhythm and metabolism of skeletal muscle. Expression levels of the core clock genes *Per2* and *Cry2* were significantly upregulated in SPF mice treated with metronidazole, with a marginal downregulation in *Bmal1*, suggesting that the host muscle peripheral clock genes are fine-tuned by the gut microbiota and that dysbiosis affects this regulation process. In GF mice, we observed a microbiota-independent effect of metronidazole on muscle, which interestingly contrasted with the effect seen with microbiota.

The clock-controlled genes *Dbp* and *E4BP4* are critical in determining period lengths in rat-1 fibroblasts and act as positive and negative regulators of transcriptional activity, respectively [40]. Our results show an increase in *Dbp* expression level that was not significant because of the large error limits among the metronidazole-treated SPF mice as compared to nontreated controls. However, we also found a significant increase in expression level of *E4BP4* in the SPF mice treated with metronidazole, although the relevance of this observation remains to be elucidated. Skeletal muscle–specific *Bmal1* ablation in mice results in decreased glucose uptake and oxidation, with increased *Pdk4* expression causing a switch toward increased lipid metabolism in the muscles of these mice [41]. Our results demonstrate a similar effect with metronidazole treatment in SPF mice, indicating that these mice might undergo increased use of lipids for energy metabolism.

Both *adiponectin* and *PPARγ* were significantly upregulated in metronidazole-treated SPF mice. We speculate that these effects might enhance fatty acid uptake and insulin sensitivity of skeletal muscle. Furthermore, *FoxO* regulates adiponectin signaling through *AdipoR,* which is critical in regulating lipid and glucose homeostasis and reducing oxidative stress [42].

Our results do not rule out the alternate possibility of changes in the gut mucosal barrier leading to skeletal muscle atrophy because of poor digestion and nutrient absorption, which remains to be investigated. Interestingly, malnutrition in children has been associated with an increase in *Proteobacteria* [43]. In addition, a limitation of this study is that the tissue samples were harvested in a single time window (zeitgeber time (ZT) 4–6). However, this time point was chosen based on our finding that in GF mice, the strongest alteration in the expression of clock and clock effector genes was observed at ZT6 [19]. Furthermore, it is likely that impaired peripheral nerve activity might be accompanied by muscle incoordination, leading to altered expression of muscle clock genes. Although further studies are required to elucidate the detailed molecular mechanism by which the metronidazole-mediated alteration of gut microbiota composition modulates skeletal muscle chronometabolism, the observed skeletal muscle atrophy is likely the result of disturbed expression of both neurogenic atrophy genes and protein degradation genes.

To conclude, metronidazole causes gut dysbiosis with increased susceptibility to colonization of *Proteobacteria* and enrichment of *Erysipelotrichales*. Importantly, metronidazole causes skeletal muscle atrophy and modulates muscle chronometabolism. These data support a role for the observed side effect of metronidazole-mediated peripheral neuropathy in human patients.

## 4. Materials and Methods

### 4.1. Animals

Wild-type (WT; C57Bl/6J) mice were maintained at the SingHealth Experimental Medicine Centre (SEMC), Singapore. The mice were inbred and housed in the SPF or GF facility of the SEMC. All mice were housed in microisolator units of 5 males per cage and fed *ad libitum* with food and water. The SPF WT mice were fed a standard irradiated chow diet and the GF mice a standard autoclaved chow diet. All mice were maintained on a 12:12 h light and dark cycle. All mice were euthanized using CO_2_ gas at ZT 4–6, where ZT0 is 07:30 and ZT12 is 19:30 at the start of the light and dark periods, respectively. The animal work was approved and performed in accordance with the relevant guidelines and regulations of the Institutional Animal Care and Use Committee (IACUC Reference No. 2015/SHS/1133), SingHealth Experimental Medicine Centre, Singapore.

### 4.2. Antibiotic Metronidazole Treatment

Adult male WT SPF mice at 6–7 months of age and GF mice at 2 months of age were administered 1 g/L metronidazole (M-840-100; Gold Biotechnology, St. Louis, MO, USA) in drinking water for 4 weeks. Control WT SPF and GF mice received no metronidazole in drinking water. The drinking water was changed twice weekly to compensate for the effects of the half-life of the drug. 

### 4.3. Mouse Fecal Pellet and Tissue Harvest

Mouse fecal pellets were collected before euthanization, snap frozen in liquid nitrogen, and stored at −80 °C until further analysis of the microbiota composition by 16s rRNA gene sequencing. The hind limb tibialis anterior, extensor digitorum longus, gastrocnemius, soleus, and quadriceps muscles were harvested from both legs, weighed, snap frozen in liquid nitrogen, and stored at −80 °C until further analysis.

### 4.4. 16S rRNA Gene Sequencing of the Fecal Microbiota Population

Bacterial genomic DNA extraction, amplification, and 16S rRNA gene sequencing of the metronidazole-treated SPF WT mice was carried out using a FastDNA^TM^ SPIN kit according to the manufacturer’s protocol (MP Biomedical, Aurora, CT, USA) by Chunlab Inc., Seoul, South Korea [44]. Briefly, the DNA was first amplified using barcoded primers specific to the V1 and V3 regions of the bacterial 16S rRNA genes. Sequences were then sorted out by their respective unique barcodes, and low-quality reads were removed. Bacterial 16S rRNA gene sequencing of 1 μg of PCR amplified product of each sample was done using the 454 GS FLX Titanium Sequencing System (Roche, Branford, FL, USA). Sequence reads were identified using the EZBioCloud Genome Database. Fecal microbial composition, diversity, and clustering were analyzed using the CLcommunity bioinformatics software provided by Chunlab Inc., Seoul, Korea.

### 4.5. Histology

The left hind leg tibialis anterior muscle was OCT (optimum cutting temperature) embedded in liquid nitrogen–cooled isopentane and stored at −80 °C. Serial transverse cryosections 10 μm thick were obtained from the mid-belly region using a Leica cryostat machine. The sections were stained with hematoxylin and eosin, cleared, and mounted in DPX (distyrene plasticiser xylene) mounting compound. Representative pictures were taken with the brightfield 10× objective of a Leica microscope and analyzed with Image J software (National Institutes of Health, Bethesda, MD, USA).

### 4.6. RNA Isolation and RT-qPCR Analysis

Total RNA was isolated from the gastrocnemius muscle using Trizol reagent and a PureLink^®^ RNA Mini Kit (Life Technologies, Carlsbad, CA, USA) according to the manufacturer’s instructions. cDNA was synthesized from 1 μg of RNA using iScript Reverse Transcription Supermix (Bio-Rad, Hercules, CA, USA). Real-time quantitative PCR was performed with KAPA SYBR FAST qPCR kits (KAPA Biosystems, Wilmington, MA, USA). The oligonucleotides (Supplementary Table S1) were ordered from Integrated DNA Technologies, Singapore. The relative mRNA levels were quantified using the ABI StepOne Plus RT-qPCR System in accordance with KAPA Biosystem cycling conditions. All amplified PCR products were calculated based on comparative delta-Ct method and normalized to endogenous glyceraldehyde-3-phosphate dehydrogenase mRNA levels.

### 4.7. Statistical Analysis

Statistical differences between groups were determined using unpaired two-tailed student’s *t*-tests, and the results were considered significant at *p* < 0.05 (*), *p* < 0.01 (**), or *p* < 0.001 (***). Data are expressed as mean ± standard error of the mean (SEM).

## Supplementary Materials

Supplementary materials can be found at http://www.mdpi.com/1422-0067/19/8/2418/s1.

## Abbreviations

Clock	Circadian locomotor output cycles kaput
Bmal1	Brain and muscle ARNT-like 1
Per	Period
Cry	Cryptochrome
Ror	RAR-related orphan receptor
E4BP4	E4 promotor binding protein 4
Dbp	D site binding protein
Akt	Akt serine/threonine kinase
MuRF1	Muscle RING-finger protein 1
MyoD	Myogenic differentiation
Mul1	Mitochondrial E3 ubiquitin protein ligase 1
LC3-1	MAF1 light chain 3–like protein 1
Ppar	Peroxisome proliferator activated receptor
Pgc-1α	Ppar gamma coactivator 1 alpha
Sirt1	Sirtuin 1
AMPK	AMP-activated protein kinase
AdipoR	Adiponectin receptor
Fto	Fat and obesity–associated protein
