# Supplementary material for: Metronidazole Causes Skeletal Muscle Atrophy and Modulates Muscle Chronometabolism
Source: Int J Mol Sci. 2018 Aug 16;19(8):2418. doi: 10.3390/ijms19082418 (PMC6121908; doi:10.3390/ijms19082418)
Supplement: Supplementary file 1 [file ijms-19-02418-s001.pdf]

International Journal of *Molecular Sciences*
MDPI

## Supplementary Materials

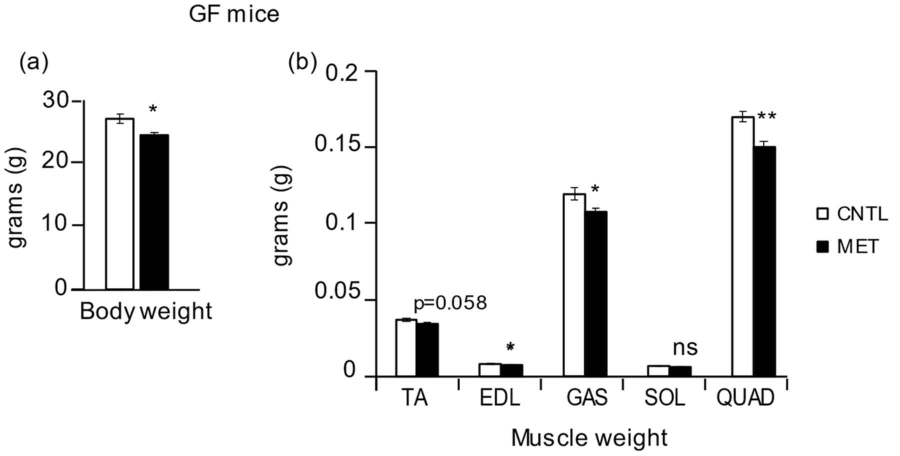


**Figure S1.** Metronidazole causes decreased body weight and hind limb muscle weight in germ-free mice. (**a**) Metronidazole treatment of GF mice resulted in a significant decrease in body weight compared to nontreated controls. $N = 5$ mice per group. **(b)** Absolute hind limb muscle weights of tibialis anterior (TA), extensor digitorum longus (EDL), gastrocnemius (GAS), soleus (SOL), and quadriceps (QUAD) muscles of metronidazole-treated GF mice (MET) compared to nontreated controls (CNTL). $N = 5$ mice per group. Data presented as means ± SEM. Asterisks indicate statistically significant differences (*, $p < 0.05$; **, $p < 0.01$; ns, nonsignificant with student's $t$ test).

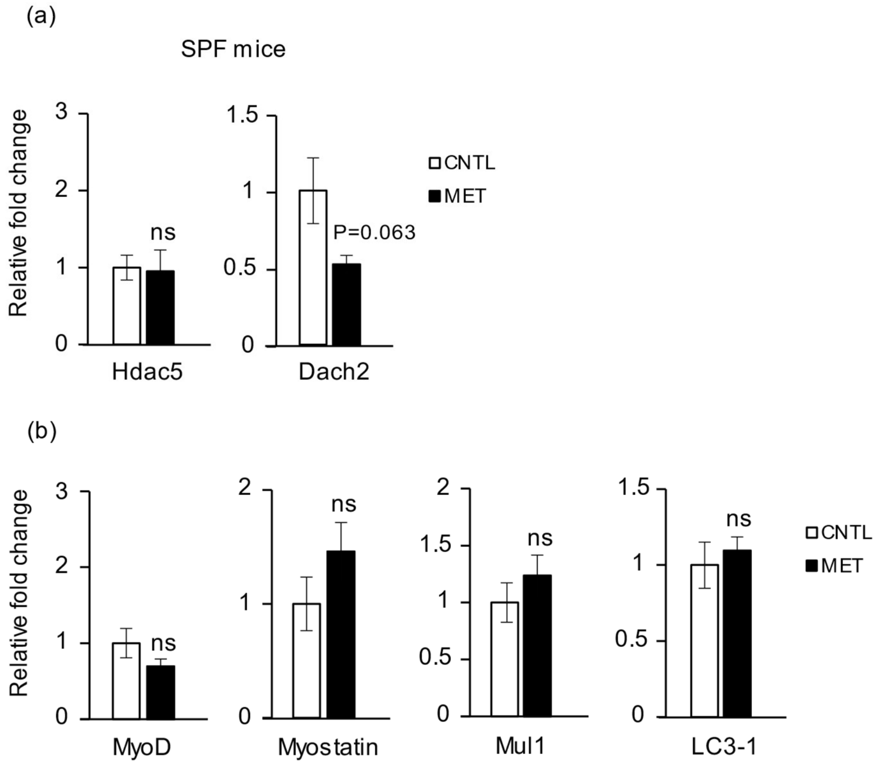

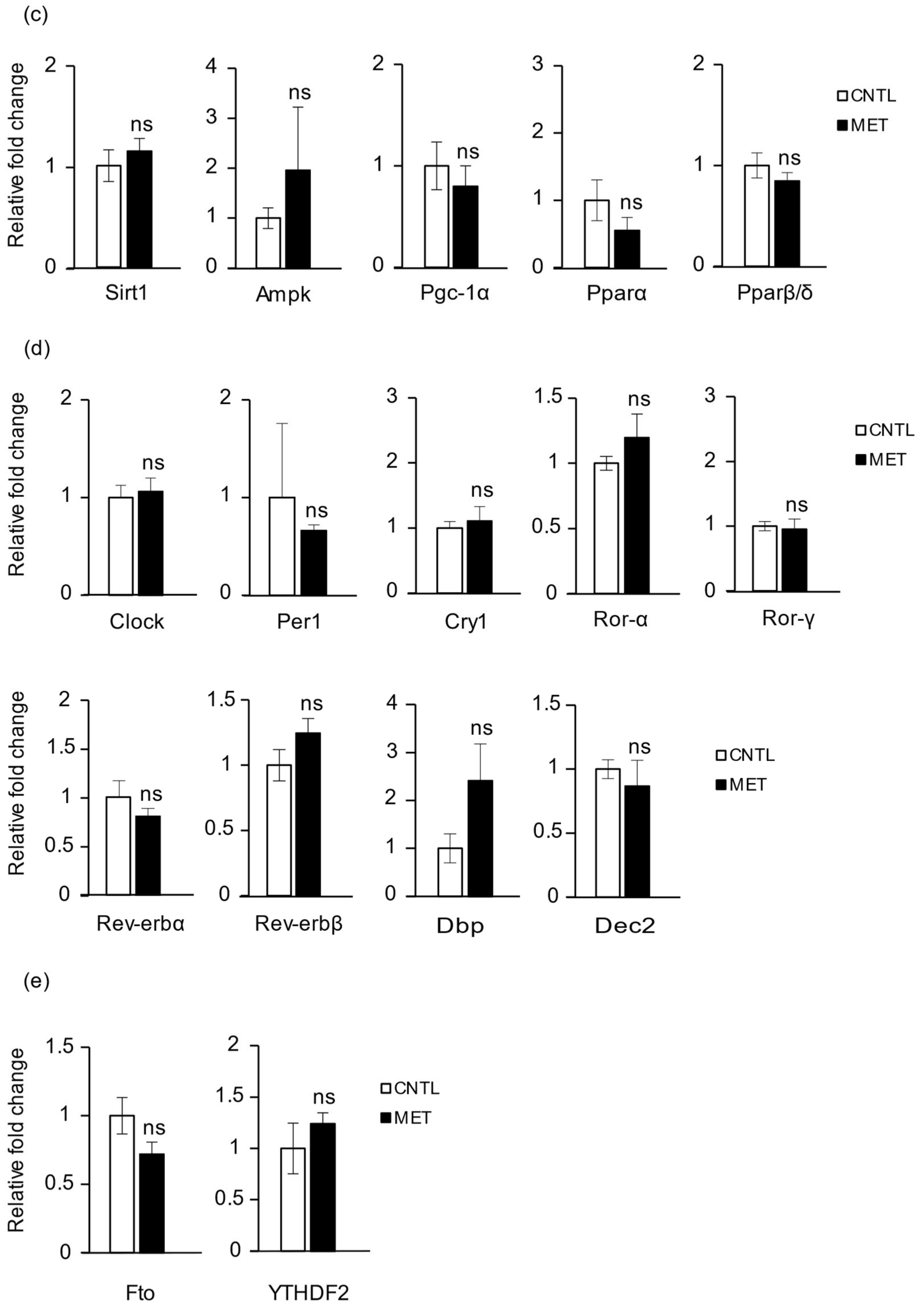


**Figure S2.** Metronidazole-treated SPF mice muscle mass determinants, autophagy markers, circadian metabolic regulators, and RNA epigenetics. Real-time quantitative PCR analysis of gene expression in gastrocnemius muscle of metronidazole-treated and nontreated SPF mice. (**a**) Neurogenic atrophy markers *Hdac5* and *Dach2*; (**b**) muscle mass determinants *MyoD* and *myostatin*, and autophagy markers *Mul1* and *LC3-1*; (**c**) circadian metabolic regulators *Sirt1*, *Ampk*, *Pgc-1α*, *PPARα*, and *PPARβ/δ*; (**d**) clock genes *Clock*, *Per1*, *Cry1*, *Ror-α*, *Ror-γ*, *Rev-erbα*, and *Rev-erbβ*, and clock effector genes *Dbp* and *Dec2*; (**e**) mRNA demethylase *Fto* and YTH $N^6$-methyladenosine RNA binding protein 2 gene *YTHDF2*. *N* = 5 mice per group. Data presented as means ± SEM (ns, nonsignificant with student's *t* test).

**Table S1.**

| Serial No. | Primer | Species | Forward primer (5'→3') | Reverse primer (5'→3') | NCBI reference |
|---|---|---|---|---|---|
| 1 | Adiponectin | Mouse | TGTTGGAATGACAGGAGCTGAA | CACACTGAAGCCTGAGCGATAC | NM_009605 |
| 2 | Ampk | Mouse | GCCCTTCCCGCAAGATGTTAT | TCAGCAGGGGTGGACTTTAAT | NM_01310480 |
| 3 | Atrogin1 | Mouse | GAGTGGCATCGCCCAAAAGA | TCTCCATCCGATACACCCACA | NM_026346 |
| 4 | Bmal1 | Mouse | ACAGTCAGATTGAAAAGAGGCG | GCCATCCTTAGCACGGTGAG | NM_001357070 |
| 5 | Clock | Mouse | ACCGTAGCAGGTTTATGGGAATG | TGGTGTCCACACAATAGGCAAGA | NM_001305222 |
| 6 | Cry1 | Mouse | CAGACTCTCGTCAGCAAGATG | CAAACGTGTAAGTGCCTCAGT | NM_007771 |
| 7 | Cry2 | Mouse | GCGTCTGTTTGTAGTCCGGG | TCCCAAAGGGTTCAGAGTCATA | NM_009963 |
| 8 | Dach2 | Mouse | TCTCCCTCGCAGATGGATCA | TGACTGGGATTTGAGCTGGT | NM_001142570 |
| 9 | Dbp | Mouse | AAGAAGGCAAGGAAAGTCCA | TGTACCTCCGGCTCCAGTA | NM_016974 |
| 10 | Dec2 | Mouse | GCGAGACGATACCAAGGATAC | TCAGATGTTCGGGCAGTAAA | NM_001271768 |
| 11 | E4BP4 | Mouse | ACGGACCAGGGAGCAGAAC | GGACTTCAGCCTCTCATCCATC | NM_017373 |
| 12 | FoxO1 | Mouse | ACCAGGAGAAGCTCCCAAGT | AATGTAGCCTGCTCACTAACTCTTA | NM_019739 |
| 13 | FoxO3 | Mouse | GCAAGCCGTGTACTGTGGA | CGGGAGCGCGATGTTATCC | NM_019740 |
| 14 | Fto | Mouse | CTCCTTGAGGAGCTTGAAGACA | CCATGCTTATGCAGTGTGAGAA | NM_011936 |
| 15 | Gapdh | Mouse | TGGCCTTCCGTGTTCCTAC | GAGTTGCTGTTGAAGTCGCA | NM_001289726 |
| 16 | Hdac4 | Mouse | CTGGGGCGCCTGACG | CTCACTGGCAGCGTCTTGTA | NM_207225 |
| 17 | Hdac5 | Mouse | TGTTGCCACTCAAGAGCACA | CACAATGATGAAGCCCAAAGGG | NM_001077696 |
| 18 | LC3-1 | Mouse | GCGAGTTGGTCAAGATCATCCG | TGGACACACTCACCATGCTGTG | NM_025735 |
| 19 | Mettl3 | Mouse | ATCCAGGCCCATAAGAAACAG | CTATCACTACGGAAGGTTGGG | NM_019721 |
| 20 | Mettl14 | Mouse | CTGAGAGTGCGGATAGCATTG | GAGCAGATGTATCATAGGAAGCC | NM_201638 |
| 21 | Mul1 | Mouse | AGGGCATTCTTTCAGAAGCA | GGGGTGGAACTTCTCGTACA | NM_026689 |
| 22 | MuRF1 | Mouse | GTGTGAGGTGCCTACTTGCTC | GCTCAGTCTTCTGTCCTTGGA | NM_001039048 |
| 23 | MyoD | Mouse | GACAGGGAGGAGGGGTAGAG | TGCTGTCTCAAAGGAGCAGA | NM_010866 |
| 24 | Myogenin | Mouse | ACTCCCTTACGTCCATCGTG | CAGGACAGCCCCACTTAAAA | NM_031189 |
| 25 | Myostatin | Mouse | CAGCCTGAATCCAACTTAGG | TCGCAGTCAAGCCCAAAGTC | NM_010834 |
| 26 | Pdk4 | Mouse | ACCCTACGGATCCTAACCACC | TCACAGGCATTTTCTGAACCAAAG | NM_013743 |
| 27 | Per1 | Mouse | GAATTGGAGCATATCACATCCGA | CCCGAAACACATCCCGTTTG | NM_001159367 |
| 28 | Per2 | Mouse | AAAGCTGACGCACACAAAGAA | ACTCCTCATTAGCCTTCACCT | NM_011066 |
| 29 | Pgc-1α | Mouse | AAACTTGCTAGCGGTCCTCA | TGGCTGGTGCCAGTAAGAG | NM_008904 |
| 30 | Pparα | Mouse | TACTGCCGTTTTCACAAGTGC | AGGTCGTGTTCACAGGTAAGA | NM_011144 |
| 31 | Pparβ/δ | Mouse | CGGCAGCCTCAACATGG | AGATCCGATCGCACTTCTCATAC | NM_011145 |
| 32 | Pparγ | Mouse | TGTGGGGATAAAGCATCAGGC | CCGGCAGTTAAGATCACACCTAT | NM_011146 |
| 33 | Rev-erbα | Mouse | CTTCCGTGACCTTTCTCAGA | CAGCTCCTCCTCGGTAAGTG | NM_145434 |
| 34 | Rev-erbβ | Mouse | CGCCATGGAGCTGAACG | GACAAGAGGCAGGGCTGGA | NM_011584 |
| 35 | Ror-α | Mouse | ACCGTGTCCATGGCAGAAC | TTTCCAGGTGGGATTTGGAT | NM_001289916 |
| 36 | Ror-β | Mouse | GGCAGACCCACACCTACGA | CAGAGCCTCCCTGGACTTG | NM_001289921 |
| 37 | Ror-γ | Mouse | TCTACACGGCCCTGGTTCT | ATGTTCCACTCTCCTCTTCTCTTG | NM_001293734 |
| 38 | Sirt1 | Mouse | CGGCTACCGAGGTCCATATAC | ACAATCTGCCACAGCGTCAT | NM_001159589 |
| 39 | Tef | Mouse | GCCGAGCTTCGCAAGGA | ACAGGTTACAAGGGCCCGTACT | NM_153484 |
| 40 | YTHDF2 | Mouse | TAGCCAACTGCGACACATTC | CACGACCTTGACGTTCCTTT | NM_145393 |
